# Hospital Reform

**Published:** 1897-10-16

**Authors:** 


### HOSPITAL REFORM.

Dr. T. Garrett Horder, hon. secretary of the Hospital Reform Association, writes: I have the honour of informing you that a conference has been arranged to take place at St. Martin's Hall, Charing Cross Road, London, on Thursday, October 21st, at 4.30 p.m. The Right Honourable the Earl of Stamford has kindly consented to preside, and the following gentlemen have promised to attend and speak: Sir William Henry Broadbent, Bart., M.D., Sir Henry Burdett, K.C.B., Dr. Isambard Owen, Mr. C. S. Loch, Mr. Victor Horsley, F.R.S., Dr. Lovell Drage, and Mr. Timothy Holmes, F.R.C.S. It has been arranged to discuss the following question: The Administration of Medical Relief in the Out-Patient and Casualty Departments of Hospitals and Dispensaries. The Hospital Reform Association begs to suggest that the conference should deal more especially with the following points: (1) Subscribers' Letters; (2) Patients' Payments; (3) Limitation of number of new cases; (4) Means to to be employed to restrict relief to fit and proper cases. It is to be hoped that the subject may be discussed (1) by hospital managers, (2) by members of the medical staffs, (3) by general practitioners, and (4) by representatives of supporters of hospitals. The conference will adjourn at 6.30 and meet again at 8 p.m. The hon. secretary will be glad to receive donations towards defraying the expenses of the meeting.

